# Addiction Consult Service and Inpatient Outcomes Among Patients with OUD

**DOI:** 10.1007/s11606-024-08837-0

**Published:** 2024-08-13

**Authors:** Andrea Jakubowski, Sumeet Singh‑Tan, Kristine Torres‑Lockhart, Tiffany Lu, Julia Arnsten, William Southern, Shadi Nahvi

**Affiliations:** 1 Division of General Internal Medicine, Department of Medicine, Montefiore Medical Center, Albert Einstein College of Medicine, Bronx, NY USA; 2 Division of Hospital Medicine, Department of Medicine, Montefiore Medical Center, Albert Einstein College of Medicine, Bronx, NY USA; 3 Department of Psychiatry and Behavioral Sciences, Montefiore Medical Center, Albert Einstein College of Medicine, Bronx, NY USA; 4 Department of Epidemiology and Population Health, Montefiore Medical Center, Albert Einstein College of Medicine, Bronx, NY USA

**Keywords:** medications for opioid use disorder, substance use disorder, addiction consult.

## Abstract

**Background:**

Despite rising hospitalizations for opioid use disorder (OUD), rates of inpatient medications for OUD (MOUD) initiation are low. Addiction consult services (ACSs) facilitate inpatient MOUD initiation and linkage to post-discharge MOUD, but few studies have rigorously examined ACS OUD outcomes.

**Objective:**

To determine the association between ACS consultation and inpatient MOUD initiation, discharge MOUD provision, and post-discharge MOUD linkage.

**Design:**

Retrospective study comparing admissions that received an ACS consult and propensity score–matched historical control admissions.

**Subjects:**

One hundred admissions with an OUD-related diagnosis, of patients not currently receiving MOUD who received an ACS consult, and 100 matched historical controls.

**Intervention:**

Consultation from an interprofessional ACS offering expertise in MOUD initiation and linkage to post-discharge MOUD.

**Main Measures:**

The primary outcome was inpatient MOUD initiation (methadone or buprenorphine). Secondary outcomes were inpatient buprenorphine initiation, inpatient methadone initiation, discharge prescription for buprenorphine, linkage to post-discharge MOUD (buprenorphine prescription within 60 days and new methadone administration at a methadone program within 30 days after discharge), patient-directed discharge, 30-day readmission, and 30-day emergency department (ED) visit.

**Key Results:**

Among 200 admissions with an OUD-related diagnosis, those that received an ACS consultation were significantly more likely to have inpatient MOUD initiation (OR 2.57 [CI 1.44–4.61]), inpatient buprenorphine initiation (OR 5.50 [2.14–14.15]), a discharge prescription for buprenorphine (OR 17.22 [3.94–75.13]), a buprenorphine prescription within 60 days (22.0% vs. 0.0%, *p* < 0.001; of those with inpatient buprenorphine initiation: 84.6% vs. 0.0%), and new methadone administration at a methadone program within 30 days after discharge (7.0% vs. 0.0%, *p* = 0.007; of those with inpatient methadone initiation: 19.4% vs. 0.0%). There were no significant differences in other secondary outcomes.

**Conclusions:**

There was a strong association between ACS consultation and inpatient MOUD initiation and linkage to post-discharge MOUD. ACSs promote the delivery of evidence-based care for patients with OUD.

**Supplementary Information:**

The online version contains supplementary material available at 10.1007/s11606-024-08837-0.

## INTRODUCTION

The US opioid overdose crisis continues unabated, with opioid overdose deaths more than quadrupling from 1999 to 2021.^1^ Methadone and buprenorphine, the two medications for opioid use disorder (MOUD) with the strongest evidence base,^2^ are safe and effective, and reduce mortality by more than half when continued long-term.^3^ Methadone and buprenorphine are also associated with reduced risk of hepatitis C and HIV transmission,^4,5^ opioid overdose mortality,^6^ and improvements in quality of life.^7^ Yet, most people with OUD do not receive these lifesaving medications.^8^

The hospitalization rate for opioid use disorder (OUD) more than tripled from 1998–2000 to 2015–2016,^9^ with corresponding increases in the incidence of acute hepatitis C^10^ and endocarditis^11^ and hospitalizations for opioid overdoses.^12^ With this rise in hospitalizations, the acute care setting has emerged as an important “touchpoint” in addressing opioid use disorder (OUD).^13^ Furthermore, people with substance use disorders (SUDs) have low engagement in primary medical care^14^ and are more likely to be hospitalized than people without SUDs.^15^ Thus, hospitalizations present crucial opportunities to offer MOUD, the gold standard of OUD treatment.^2^

Initiating MOUD in the hospital can improve patient outcomes during hospitalization by treating opioid withdrawal, with untreated withdrawal representing an important reason for patient-directed discharge.^16^ Inpatient MOUD initiation is also associated with engagement in MOUD after hospital discharge.^17^ Long-term outpatient MOUD engagement is essential to reducing OUD-related mortality.^3^ Yet, one study in the Veterans Health Administration found that only 2% of over 12,000 hospitalized patients with OUD received MOUD with linkage to post-discharge MOUD.^18^ Another study found that only 16% of patients received follow-up OUD care after presenting to the emergency department with an opioid overdose.^19^ There are numerous systemic reasons for low rates of inpatient MOUD initiation, including the historical requirement for a buprenorphine prescribing waiver,^20^ limited training in SUDs in undergraduate and graduate medical education,^20^ and insufficient support for linkage to post-discharge MOUD.^21,22^

Addiction consult services (ACSs) have been developed to address the need for evidence-based treatment of SUDs, including promoting inpatient MOUD initiation and linkage to post-discharge MOUD. ACSs show promise for increasing inpatient MOUD initiation and linkage to long-term treatment,^23^ but many studies examining the association between ACS consultation and clinical outcomes have been limited by the lack of a rigorous comparison group or have evaluated ACSs more generally, without specifically examining outcomes among patients with OUD.^23^

Our objective in this study was to rigorously evaluate the association between ACS consultation and inpatient MOUD initiation. We also evaluated the association between ACS consultation and discharge prescription for buprenorphine, linkage to post-discharge MOUD, patient-directed discharge, 30-day readmission, and 30-day post-discharge emergency department (ED) visits.

## METHODS

### Setting and Patient Population

Weiler hospital is one of three academic adult hospitals that make up Montefiore Medical Center in the Bronx, NY. The Bronx is one of the nation’s poorest urban counties^24^ with a high burden of opioid-related hospitalizations and overdose deaths.^25,26^ Montefiore is a major provider of MOUD in the Bronx; six of the 15 opioid treatment programs in the Bronx are Montefiore affiliated,^27^ and Montefiore’s buprenorphine treatment program has served over 1300 patients in six locations since 2005.^28^ Patients were eligible for inclusion in the study if they (1) were admitted following the launch of the ACS (hereafter the “consult-available” time period, April 27, 2021 through December 20, 2021) or prior to the launch of the ACS (hereafter, the “consult-unavailable” time period, April 27, 2019, through December 20, 2019), (2) had a primary or secondary opioid-related diagnosis at discharge based on International Classification of Diseases (ICD-10) codes (Appendix), and (3) did not have evidence of current treatment with methadone or buprenorphine. The time periods for the study were chosen to include similar calendar days and to exclude periods when COVID-19 was surging in the inpatient population. Evidence of current treatment with methadone was defined as receipt of methadone at a Montefiore Medical Center methadone program within 60 days before admission, or a first dose of methadone greater than 40 mg during the admission. Evidence of current treatment with buprenorphine was defined as a prescription in their electronic health record within 60 days before admission, or a first dose of buprenorphine greater than 2 mg during the admission. We chose 2 mg of buprenorphine because the clinical guideline in use during the study period recommended initiating with either 2 or 4 mg and common practice was to initiate with 2 mg. During the study period, methadone was used more often than buprenorphine for withdrawal management if patients did not wish to continue MOUD after discharge. During the consult-unavailable time period, patients initiating methadone in the hospital generally were responsible for finding a methadone program on discharge if they were interested. Because some admissions (9.5%) represented subsequent admissions for patients with multiple admissions, and each individual admission was a new opportunity for ACS consultation, we used *admission* rather than *patient* as the unit of analysis.

### Intervention

The ACS launched at Weiler Hospital on April 20, 2021. The ACS is an interprofessional hospital-based consult service^29^ that assists with substance use withdrawal management, SUD treatment, patient-centered harm reduction counseling, peer engagement, discharge planning, and linkage to outpatient care for patients with SUDs. The ACS consists of a multidisciplinary team including a board-certified addiction medicine attending physician, an addiction medicine fellow, a peer advocate, and other medical trainees. It works in close collaboration with nursing, social work, psychiatry, and pharmacy departments.

The ACS clinicians counsel patients with OUD about MOUD options and provide the primary team with recommendations for initiation and titration of MOUD. The consult service primarily recommends methadone or buprenorphine, as these are the MOUD with the strongest evidence base.^2^ The service provides daily evaluation and recommendations for the care of patients during admission. Before discharge, the ACS links patients with a post-discharge appointment for continued buprenorphine or methadone treatment at outpatient treatment programs affiliated with the academic medical center, depending on patient preference. The peer advocate serves as a continued point of contact after hospital discharge.

### Study Design

We evaluated the effect of an ACS consultation on patient-care outcomes using electronic health record (EHR) data. To address the potential for confounding by indication, wherein patients who received an ACS consult would have differing demographic, clinical, and hospitalization characteristics than those who did not, we used a propensity score–matched historical control design. Using this design, we created a control group with a similar likelihood of being consulted by the ACS if it were available.^30^ First, we identified the study-eligible population, consisting of two cohorts: (1) the consult-available cohort consisted of admissions during the consult-available time period and (2) the consult-unavailable cohort consisted of admissions during the consult-unavailable time period. Then, to create the matched study sample, each admission from the consult-available cohort who received an ACS consult was matched with a historical control admission from the consult-unavailable cohort using a propensity score matching protocol. We follow STROBE reporting guidelines for cohort studies.^31^ This study was determined to be exempt by the Albert Einstein College of Medicine Institutional Review Board.

### Data Sources

Data were extracted by WS and AJ from two EHRs: (1) the EHR used by Montefiore Medical Center for all inpatient and outpatient medical visits, including for primary-care-based buprenorphine treatment, and (2) the EHR used by Montefiore outpatient SUD treatment programs, where all outpatient methadone dosing data is maintained. All data was maintained confidentially in a password-protected folder on a secure server. Only data from the Montefiore health system were available.

### Measures

#### Outcomes

The primary outcome was inpatient treatment with MOUD, hereafter called inpatient MOUD initiation. This was a dichotomous outcome defined as a composite of treatment with methadone (oral formulation) or buprenorphine (sublingual formulation) during admission. We chose not to include naltrexone in our analyses because methadone and buprenorphine, but not naltrexone, have been shown to reduce overdose and mortality risk;^2,32,33^ therefore, the ACS preferentially initiates methadone and buprenorphine. We separated the composite outcome of inpatient MOUD initiation into its methadone and buprenorphine components as secondary outcomes. Other secondary outcomes included discharge prescription for buprenorphine and linkage to post-discharge MOUD (buprenorphine prescription within 60 days after discharge, and new methadone administration at a methadone program within 30 days after discharge), patient-directed discharge, 30-day readmission, and 30-day post-discharge emergency department (ED) visit. We chose 60 days for buprenorphine linkage (like in Suzuki et al., 2015)^34^ because it was common practice for the ACS to recommend discharge prescriptions of 2 or more weeks and proactively contact patients who missed linkage appointments to reschedule them (even multiple times), which could result in a linkage appointment greater than 30 days after hospital discharge. Discharge prescription for buprenorphine was a dichotomous outcome defined as a prescription for buprenorphine ordered at discharge. Buprenorphine prescription within 60 days after discharge was a dichotomous outcome defined as an additional prescription for buprenorphine in the medical record within 60 days of discharge. Methadone administration was a dichotomous outcome defined as having any records of methadone administration at a methadone program affiliated with the academic medical center within 30 days after discharge. Patient-directed discharge was a dichotomous outcome defined by a disposition code of discharge against medical advice or elopement in the medical record. Thirty-day readmission and post-discharge ED visit were dichotomous outcomes defined as a hospital readmission or ED visit within the Montefiore Medical Center during the 30 days after discharge.

#### Covariates

To create propensity-matched cohorts, we examined the demographic, clinical, and hospitalization characteristics for each admission. Demographic characteristics included age, sex, race/ethnicity (non-Hispanic/Latinx Black/African American, non-Hispanic/Latinx white, Hispanic/Latinx and non-Hispanic/Latinx other/unknown), and insurance (private, Medicaid, Medicare, and other). We created five categories of ICD-10 opioid-related diagnosis codes: (1) Adverse effect of opioid; (2) Opioid abuse and Opioid-induced disorders; (3) Opioid Use or Dependence with current complication (intoxication OR withdrawal OR psychiatric effect); (4) Opioid Use OR Opioid Dependence without current complication; (5) Poisoning (see the Appendix for a complete list of diagnoses by category). Some admissions had multiple diagnoses falling under more than one diagnosis category. To assign a single diagnosis category to each admission to create matched cohorts, we prioritized categories numerically with “(1) Poisoning” assigned highest priority, and “(5) Opioid Use OR Dependence (without indication of current complication)” assigned lowest priority. Other clinical characteristics included comorbid substance use disorder (including cocaine, sedative/hypnotic, stimulant, or alcohol use by ICD-10 diagnosis code); diagnosis of liver disease; Charlson comorbidity index;^35^ and the number of admissions and ED visits in the year prior to admission. The hospitalization characteristic was admission to the intensive care unit (ICU) from the ED (dichotomous).

### Creation of Matched Cohorts

For each admission that received an ACS consult, a propensity score–matched control admission from the consult-unavailable time period was sought. First, a propensity score model in the unmatched consult-available cohort was created to calculate the propensity to receive an ACS consult using a non-parsimonious model-building strategy, which included all demographic, clinical, and admission-level covariates. Next, the β-coefficients from the propensity score model were used to calculate propensity scores for all admissions in the unmatched consult-unavailable cohort. Finally, these cohorts were matched using a nearest neighbor protocol with a caliper width of 0.05 of the standard deviation of the logit of the propensity score. The matching protocol was repeated using different random sorts which yielded identical results. If balance was not achieved in all covariates after matching, models were adjusted for the unbalanced covariates.

### Analysis

First, the demographic, clinical, and hospitalization characteristics of the matched cohorts were compared using *t*-tests and chi-square tests as appropriate. Next, to determine associations between an ACS consult and the outcomes (inpatient MOUD initiation, discharge prescription for buprenorphine, linkage to post-discharge MOUD, patient-directed discharge, 30-day readmission, and 30-day post-discharge ED visits), univariable and multivariable logistic regression models were used. Adjustment variables were included in the multivariable models if bivariate testing yielded a *p*-value ≤ 0.2. In a post hoc sensitivity analysis, we adjusted for all variables in which the SMD was ≥ 0.1, which did not alter the results appreciably. Because the unit of analysis was hospital admission, all regressions used cluster-robust standard errors to account for the clustering of multiple admissions within individual patients. All analyses were completed using Stata 17 software (StataCorps. 2021).

## RESULTS

### Study Population

**Figure 1:**
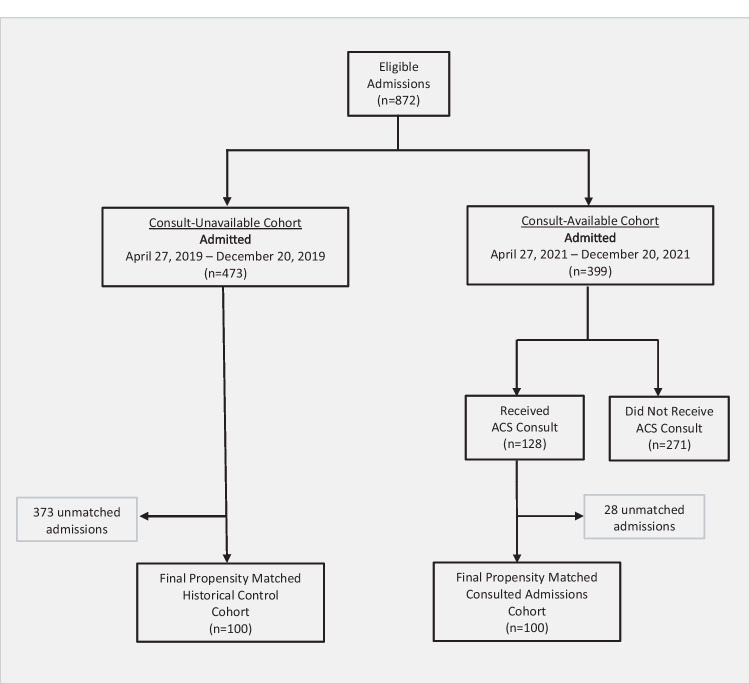
Flow diagram of propensity-matched admissions.

There were 872 admissions eligible for the study (681 unique patients), including 473 admissions in the consult-unavailable period and 399 in the consult-available period. Of the admissions in the consult-available period, 128 admissions received a consult by the ACS, and the remaining 271 did not. Of the 128 admissions receiving a consult, 100 were successfully matched with historical control admissions, yielding a final study sample of 200 admissions (181 unique patients) (Fig. 1). When compared with matched historical controls, the admissions receiving an ACS consult were less likely to have a comorbid non-opioid SUD (41% vs. 53%, *p* = 0.09) (Table 1).

**Table 1:** Baseline Characteristics of Propensity Score–Matched Admissions

	Historical control (*n* = 100)	Consulted admission (*n* = 100)	*p*-value	Standardized mean difference
Age, mean (SD)	54.1 (14.15)	55.0 (12.72)	0.61	0.07
Male sex, %	55 (55.0%)	57 (57.0%)	0.78	− 0.04
Race/ethnicity, %			0.49	− 0.22
Non-Hispanic/Latinx Black/African American	25 (25.0%)	33 (33.0%)		
Non-Hispanic/Latinx white	13 (13.0%)	15 (15.0%)		
Hispanic/Latinx	54 (54.0%)	47.47.0%)		
Non-Hispanic/Latinx other race/unavailable^*^	8 (8.0%)	5 (5.0%)		
Insurance, %			0.64	0.09
Private	7 (7.0%)	4 (4.0%)		
Medicaid	57 (57.0%)	58 (58.0%)		
Medicare	36 (36.0%)	38 (38.0%)		
ICD-10 opioid-related diagnosis, %^†^			0.82	0.01
Adverse effect of opioid	8 (8.0%)	9 (9.0%)		
Opioid abuse and opioid-induced disorders	12 (12.0%)	13 (13.0%)		
Opioid use or dependence with current complication	17 (17.0%)	18 (18.0%)		
Opioid use or dependence without current complication	47 (47.0%)	39 (39.0%)		
Poisoning	16 (16.0%)	21 (21.0%)		
Comorbid substance use disorder^§^	53 (53.0%)	41 (41.0%)	0.09	− 0.24
Charlson comorbidity index ≥ 2	68 (68.0%)	72 (72.0%)	0.54	0.09
Liver disease^†^	29 (29.0%)	35 (35.0%)	0.36	0.13
ICU admission	8 (8.0%)	9 (9.0%)	0.80	0.04
Previous ED visits (1 year)			0.47	0.05
None	31 (31.0%)	23 (23.0%)		
1–4 visits	41 (41.0%)	49 (49.0%)		
5–9 visits	14 (14.0%)	17 (17.0%)		
≥ 10 visits	14 (14.0%)	11 (11.0%)		
Previous admissions (1 year)			0.75	0.04
None	55 (55.0%)	49.0%)		
1–4 admissions	27 (27.0%)	34 (34.0%)		
5–9 admissions	11 (11.0%)	11 (11.0%)		
≥ 10 admissions	7 (7.0%)	6 (6.0%)		

^*^Includes people who are Asian, Native American/Alaskan Native, or without available race data

^†^Based on ICD-10 diagnosis code listed at discharge

^§^Includes cocaine, sedative/hypnotic, stimulant, and alcohol use disorders based on ICD-10 diagnoses

### Inpatient MOUD Initiation

Admissions with an ACS consult were more likely than controls to initiate MOUD during admission in both the unadjusted model (57.0% vs. 34%; OR 2.57 [CI 1.44–4.61]) and after adjustment for comorbid SUD (AOR 2.73 [1.51–4.93]). Consulted admissions were more likely to initiate inpatient buprenorphine (26.0% vs. 6.0%; OR 5.50 [2.14–14.15]; AOR 5.15 [2.00–13.34]). Numerically, more consulted admissions than controls initiated inpatient methadone, but this was not statistically significant (36.0% vs. 31.0%; OR 1.25 [0.69–2.29]; AOR 1.38 [0.74–2.55]).

### Secondary Outcomes

Admissions with an ACS consult had a greater likelihood of receiving a buprenorphine prescription at discharge in both unadjusted (26.0% vs. 2.0%; 17.22 [3.94–75.13]) and adjusted (AOR 16.42 [3.76–71.76]) analyses. Admissions receiving an ACS consult were also more likely to have received a prescription for buprenorphine in the 60 days after discharge (entire sample: 22.0% vs. 0.0%, *p* < 0.001; of those who had inpatient buprenorphine initiation: 84.6% vs. 0.0%) and to be admitted into a methadone program within 30 days of discharge (entire sample: 7.0% vs. 0.0%, *p* = 0.007; of those who had inpatient methadone initiation: 19.4% vs. 0.0%). Admissions with an ACS consult and matched historical controls did not have statistically significant differences in rates of patient-directed discharge in unadjusted (16.0% vs. 15.0%; OR 1.08 [0.48–2.44]]) and adjusted (AOR 1.03 [0.45–2.37)]) analyses. Additionally, there were no significant differences in rates of 30-day post-discharge ED visits (32.0% vs. 32.0%; OR 1.00 [0.55–1.83]; AOR 1.01 [0.55–1.85]) or 30-day readmissions (28.0% vs. 21.0%; OR 1.46 [CI 0.76–2.82]; AOR 1.53 [CI 0.79–2.98]) among admissions receiving a consult and those that did not (Table 2).

**Table 2:** Clinical Outcomes of Consulted Admissions and Historical Controls

	Historical control (*n* = 100)	Consulted admission (*n* = 100)	*p*-value	Odds ratio (CI) Unadjusted	Odds ratio (CI) Adjusted^*^
Inpatient medication for OUD	34 (34.0%)	57 (57.0%)	0.001	2.57 (1.44–4.61)	2.73 (1.51–4.93)
Buprenorphine	6 (6.0%)	26 (26.0%)	< 0.001	5.50 (2.14–14.15)	5.15 (2.00–13.34)
Methadone	31 (31.0%)	36 (36.0%)	0.45	1.25 (0.69–2.29)	1.38 (0.74–2.55)
Buprenorphine prescription at discharge	2 (2.0%)	26 (26.0%)	< 0.001	17.22 (3.94–75.13)	16.42 (3.76–71.76)
Buprenorphine prescription within 60 days	0 (0.0%)	22 (22.0%)	< 0.001	–	–
Admitted into methadone program within 30 days of discharge	0 (0.0%)	7 (7.0%)	0.007	–	–
Patient directed discharge	15 (15.0%)	16 (16.0%)	0.85	1.08 (0.48–2.44)	1.03 (0.45–2.37)
ED visit (30 d)	32 (32.0%)	32 (32.0%)	> 0.99	1.00 (0.55–1.83)	1.01 (0.55–1.85)
Readmission (30 d)	21 (21.0%)	28 (28.0%)	0.25	1.46 (0.76–2.82)	1.53 (0.79–2.98)

^*^Adjusted for comorbid substance use disorder

## DISCUSSION

In this study, rigorous analytic methods were used to demonstrate a robust association between ACS consultation and inpatient MOUD initiation and post-discharge MOUD linkage. Specifically, there was a strong and statistically significant association between ACS consultation and inpatient buprenorphine initiation and a non-significant positive association between ACS consultation and inpatient methadone initiation. There was a robust association between ACS consultation and post-discharge linkage to both buprenorphine and methadone. There were no associations between ACS consultation and patient-directed discharge, 30-day hospital readmission, or 30-day ED visits.

This study’s findings on MOUD initiation and linkage in this rigorously controlled sample corroborate findings from the few studies that have evaluated OUD outcomes of ACSs using a comparison group.^36,37^ Consistent with this study’s findings, two studies, both of which used a non-consulted comparison group and had small sample sizes, found differences in inpatient MOUD initiation between ACS consulted and non-consulted groups.^36,37^ We found a stronger association between ACS consultation and inpatient buprenorphine initiation than for methadone initiation, consistent with another study examining buprenorphine and methadone separately.^37^ This study’s finding that 84.6% of patients with inpatient buprenorphine initiation linked to outpatient buprenorphine is higher than what has been reported in other studies of hospital buprenorphine initiation interventions (including ACSs), ranging from 47 to 72%.^17,34,38,39^ Our finding that 19.4% of those with inpatient methadone initiation were admitted into a methadone program is lower than the percentage reported by Trowbridge et al. (76%),^38^ but there are limited data in the literature specifically on methadone linkage from the hospital. The lower percentage of methadone linkages could be due to missing data on linkages made to methadone programs outside the academic medical center. Or, it could reflect that patients initiating methadone were primarily receiving methadone for withdrawal management and not interested in continuing outpatient MOUD. Findings from this study illustrate the large unmet need for addiction expertise in acute care settings^18^ and contribute to the emerging body of literature demonstrating the importance of ACSs for promoting evidence-based OUD care.^23^^,^^29^

There were no associations between ACS consultation and other secondary outcomes of interest, including 30-day readmission and 30-day ED visits. The literature on the association between addiction consultation and acute care utilization has been mixed. While some studies have shown a negative association between consultation and ED visits and/or readmission,^40–44^ others have shown no significant association,^37,45–47^ or even an increase in acute care utilization (specifically 30-day ED visits).^47^ The sample had multiple medical comorbidities and high rates of ED and hospital utilization prior to admission. A single ACS consultation may not be able to impact the numerous medical and structural factors that influence acute care utilization.

There was no difference in patient-directed discharge between the consult-available and consult-unavailable groups. Research to date has shown mixed findings on the association between addiction consultation, MOUD receipt, and patient-directed discharge.^36,40,48,49^ Though an ACS consultation has the potential to assist in the management of withdrawal symptoms and initiation of MOUD, and, in turn, reduce patient-directed discharge, it cannot address the many social and structural factors that can drive patient-directed discharge.^50^ Given the small number of admissions with patient-directed discharge, it is also possible that our study was underpowered to detect differences between the groups.

An important strength of this study is that rigorous analytic methods and a carefully selected historical comparison group were used, enhancing generalizability. This study’s findings add to those studies on the impact of ACS that had no comparison group,^34,36,38,45,51,52^ did not use adjusted analyses,^36,37^ or used a concurrent control group.^40,46,53^ Using concurrent controls (patients who did not receive a consult while a consult service was available) risks introducing bias because there are likely important clinical differences between patients who do and do not receive consults during the same time period. We are aware of one propensity-matched analysis study of ACS outcomes, which used a historical comparison group, but only one-quarter of the sample had OUD.^47^ The use of propensity score matching also allowed for comparisons of patients from the consult-available and consult-unavailable periods with similar substance use and clinical characteristics, an important strength because the consult-available period was during the COVID-19 pandemic when opioid use increased for many patients with OUD.^54^ Another strength of this study is that we describe OUD treatment outcomes in a hospital that serves large numbers of Black and Latinx patients, reflective of the Bronx, NY, the New York City borough with the highest opioid overdose burden.^26^ In New York City and across the United States, Black and Latinx people have unequal access to buprenorphine.^55,56^ Our study suggests that ACS may help remedy this inequality. Finally, it is a strength that both buprenorphine and methadone initiation and linkage are reported, while other studies have reported exclusively on buprenorphine.^34,39,57^

This study also had limitations. This study lacked data from outside the Montefiore health system, so we would not have known if a discharged patient was linked to MOUD outside the academic medical center. This limitation is shared by most other post-hospital SUD treatment linkage studies. However, the academic medical center is a major provider of buprenorphine and methadone treatment in the Bronx, which gives us confidence that the association between consultation and linkage in our study is not spurious. Doses of buprenorphine and methadone were not examined, which may have been helpful in distinguishing between MOUD initiated for maintenance (titrated to higher doses) and MOUD initiated just for withdrawal management (lower doses). Finally, a limitation is that the outcomes of interest are short-term (i.e., linkage to outpatient MOUD, not retention in treatment; 30-day ED visits and readmissions instead of longer-term acute care utilization). Future studies could examine associations between ACS consultation and longer-term outcomes.

In conclusion, ACS consultation in an academic medical center was associated with a dramatic increase in MOUD initiation (over fivefold for buprenorphine) and linkage to MOUD following hospital discharge (with no linkages in the control period). These findings add to the body of literature demonstrating the importance of ACSs for promoting hospital MOUD initiation and post-discharge linkage to outpatient MOUD. Whenever possible, hospitals should implement interprofessional ACSs to deliver evidence-based care to patients with OUD.

## Supplementary Information

Below is the link to the electronic supplementary material.

Supplementary file1 (DOCX 286 kb)

## Data Availability

The datasets analyzed during the current study are available from the corresponding author on reasonable request.
