# Supplementary material for: Addiction Consult Service and Inpatient Outcomes Among Patients with OUD
Source: J Gen Intern Med. 2024 Aug 13;39(15):2961–9. doi: 10.1007/s11606-024-08837-0 (PMC11576704; doi:10.1007/s11606-024-08837-0)
Supplement: Supplementary file 1 — Supplementary file1 (DOCX 286 kb) [file 11606_2024_8837_MOESM1_ESM.docx]

**Appendix 1: ICD-10 codes used to define the cohort**

**Poisoning (1)**

| T40.1X1A | Poisoning by heroin, accidental (unintentional), initial encounter |
| --- | --- |
| T40.1X1D | Poisoning by heroin, accidental (unintentional), subsequent encounter |
| T40.1X3A | Poisoning by heroin, assault, initial encounter |
| T40.1X2A | Poisoning by heroin, intentional self-harm, initial encounter |
| T40.1X4A | Poisoning by heroin, undetermined, initial encounter |
| T40.3X1A | Poisoning by methadone, accidental (unintentional), initial encounter |
| T40.3X2A | Poisoning by methadone, intentional self-harm, initial encounter |
| T40.3X3A | Poisoning by methadone, assault, initial encounter |
| T40.0X1A | Poisoning by opium, accidental (unintentional), initial encounter |
| T40.0X2A | Poisoning by opium, intentional self-harm, initial encounter |
| T40.2X1A | Poisoning by other opioids, accidental (unintentional), initial encounter |
| T40.2X2A | Poisoning by other opioids, intentional self-harm, initial encounter |
| T40.2X4A | Poisoning by other opioids, undetermined, initial encounter |
| T40.4X1A | Poisoning by other synthetic narcotics, accidental (unintentional), initial encounter |
| T40.601A | Poisoning by unspecified narcotics, accidental (unintentional), initial encounter |
| T40.602A | Poisoning by unspecified narcotics, intentional self-harm, initial encounter |
| T40.603A | Poisoning by unspecified narcotics, assault, initial encounter |
| T40.4X2A | Poisoning by other synthetic narcotics, intentional self-harm, initial encounter |

**Opioid abuse and Opioid-induced disorders (2)**

| F11.121 | Opioid abuse with intoxication delirium |
| --- | --- |
| F11.120 | Opioid abuse with intoxication, uncomplicated |
| F11.122 | Opioid abuse with intoxication with perceptual disturbance |
| F11.129 | Opioid abuse with intoxication, unspecified |
| F11.188 | Opioid abuse with other opioid induced disorder |
| F11.19 | Opioid abuse with unspecified opioid induced disorder |
| F11.10 | Opioid abuse, uncomplicated |
| F11.14 | Opioid abuse with opioid induced mood disorder |
| F11.151 | Opioid abuse with opioid induced psychotic disorder with hallucinations |
| F11.159 | Opioid abuse with opioid induced psychotic disorder, unspecified |
| F11.150 | Opioid abuse with opioid-induced psychotic disorder with delusions |
| F11.288 | Opioid dependence with other opioid-induced disorder |
| F11.29 | Opioid dependence with unspecified opioid-induced disorder |
| F11.988 | Opioid use, unspecified with other opioid-induced disorder |
| F11.99 | Opioid use, unspecified with unspecified opioid-induced disorder |

**Adverse Effect (3)**

| T40.3X5A | Adverse effect of methadone, initial encounter |
| --- | --- |
| T40.0X5A | Adverse effect of opium, initial encounter |
| T40.695A | Adverse effect of other narcotics, initial encounter |
| T40.2X5A | Adverse effect of other opioids, initial encounter |
| T40.4X5A | Adverse effect of other synthetic narcotics, initial encounter |
| T40.605A | Adverse effect of unspecified narcotics, initial encounter |

**Opioid Use OR Opioid Dependence with indication of current complication (intoxication or withdrawal or psychiatric effect) (4)**

| F11.221 | Opioid dependence with intoxication delirium |
| --- | --- |
| F11.220 | Opioid dependence with intoxication, uncomplicated |
| F11.229 | Opioid dependence with intoxication, unspecified |
| F11.23 | Opioid dependence with withdrawal |
| F11.24 | Opioid dependence with opioid-induced mood disorder |
| F11.259 | Opioid dependence with opioid-induced psychotic disorder, unspecified |
| F11.282 | Opioid dependence with opioid-induced sleep disorder |
| F11.921 | Opioid use, unspecified with intoxication delirium |
| F11.929 | Opioid use, unspecified with intoxication, unspecified |
| F11.93 | Opioid use, unspecified with withdrawal |
| F11.920 | Opioid use, unspecified with intoxication, uncomplicated |
| F11.959 | Opioid use, unspecified with opioid-induced psychotic disorder, unspecified |
| F11.94 | Opioid use, unspecified with opioid-induced mood disorder |

**Opioid Use OR Dependence (without indication of current complication) (5)**

| F11.20 | Opioid dependence, uncomplicated |
| --- | --- |
| F11.21 | Opioid dependence, in remission |
| F11.90 | Opioid use, unspecified, uncomplicated |
| Z79.891 | Long term (current) use of opiate analgesic |
